# Endoscopic Findings of Gastro-Esophageal Reflux Disease in Elderly and Younger Age Groups

**DOI:** 10.3389/fmed.2021.606205

**Published:** 2021-12-10

**Authors:** Haydar Adanir, Bilge Baş, Betul Pakoz, Süleyman Günay, Hakan Camyar, Muge Ustaoglu

**Affiliations:** ^1^Department of Gastroenterology, Akdeniz University, Antalya, Turkey; ^2^Department of Gastroenterology, Antalya Training and Research Hospital, Antalya, Turkey; ^3^Faculty of Medicine, Izmir Katip Celebi University, Izmir, Turkey; ^4^Faculty of Health Sciences, Ondokuz Mayıs University, Samsun, Turkey

**Keywords:** elderly, gastro-esophageal reflux, hiatal hernia, esophagitis, younger

## Abstract

**Objective:** To determine and compare the clinical features and endoscopic findings of gastro-esophageal reflux disease (GERD) in elderly and younger age groups.

**Materials and Methods:** The clinical and endoscopic features were evaluated for all patients with GERD between January 2017 and September 2020. The criteria for inclusion were being aged over 65 and under 50 years and having an upper gastrointestinal endoscopy with reflux symptoms resistant to ppi theraphy. The exclusion criteria included prior surgery, age under 18 years, and pregnancy. The diagnosis of GERD was made according to the patients' symptoms. The SPSS 11.0 for Windows pocket program was used for statistical analysis.

**Results:** Two hundred eighty-six patients aged over 65 years and 261 patients aged below 50 years were enrolled in this study. The mean age of the older group was 68.2 ± 4.5 years and the mean age of the young group was 38 ± 7.2 years. The male/female ratio was 5/3 and 2/1 in the young and older groups, respectively. The older patients had less severe and rare typical symptoms than the young patients. However, significantly more serious endoscopic findings were noted in the older patients compared with the younger patients.

**Conclusion:** The older and young patients with GERD were predominantly male and typical reflux problems were less common in older patients with GERD. Older patients had more important endoscopic findings such as hernia, esophagitis, and cancer.

## Introduction

Reflux is the retrograde movement of stomach contents toward the distal esophagus, which can normally be observed approximately 10–15 times a day and accepted as physiological (1). Physiologic reflux may be imperceptible or produce very mild symptoms due to its short duration. However, when reflux recurs at frequent intervals during the day, occurs over longer periods, and especially occurs during sleep, it is known as gastro-esophageal reflux disease (GERD). Gastro-esophageal reflux disease is defined as the gastric contents' abnormal reflux into the esophagus, resulting in mucosal damage. A defective antireflux barrier, abnormal esophageal clearance, reduced salivary production, altered esophageal mucosal resistance, and delayed gastric emptying can be mentioned among the pathogenic abnormalities causing GERD (2). Gastro-esophageal reflux disease is most common (25%) among individuals aged 25–35 years and prevalence in the elderly is 14–20% (3). On the other hand, reflux oesophagitis is more severe in the elderly than in adult or young subjects (4). With aging, many physiologic changes occur in the gastrointestinal system (GIS), such as a decrease in the stomach's elasticity, gastric secretions, and mobility; gastric atrophy; and delayed gastric emptying (5). These are directly or indirectly related to the GIS problems seen in old age. With the relaxation of the lower esophageal sphincter, the anti-reflux mechanism is disrupted and reflux symptoms are seen. The main pathology of GERD in older patients is gastric dysmotility resulting in delayed gastric emptying. Exposure to gastric acid and the duration of this exposure is important in symptom formation. Atrophic gastritis is also more common in the elderly (6).

Hiatus hernia is the most common type of hernia with a rate of 80–90%. Due to enlargement in the esophagial hiatus and weakness in the phrenoesophageal ligament, a part of the stomach, especially the cardia part and the gastro-esophageal junction, passes into the thorax. Especially in the elderly, it is more frequent due to the loss of strength in the tissues (7). Hiatal hernias usually progress with non-specific symptoms; however, reflux symptoms are also common.

Difficulties are encountered in diagnosis and treatment due to accompanying diseases and increased drug use (polypharmacy) in old age. Medications for co-morbid illnesses, such as cardiovascular disease, pulmonary disease, depression, and hypertension, are some drugs more frequently taken by the elderly and may also decrease LES pressure (8).

Gastro-esophageal reflux disease complications are also more common and potentially severe in older patients. A wide range of complications varying from mild esophagitis to major life-threatening problems such as Barrett's esophagus (BE), esophageal cancer, and recurrent pulmonary aspiration may be seen (9, 10).

The presence of endoscopic and/or histopathologic damage to the esophagus is called reflux esophagitis. The pathogenesis of esophagitis is multifactorial, the disruption of the balance between reflux protection and reflux facilitating mechanisms is responsible for this situation. The increased sensitivity of the esophageal mucosa to acid and LES pressure reduction is important. Repetitive retrosternal pain, burning or discomfort in the epigastrium spreading through the esophagus are common clinical symptoms. Heartburn usually increases after meals and is aggravated by lying down or leaning forward. Patients may also present with dysphagia, chest pain, globus sensation, belching, and cough. Chest pain can spread to the neck, chin, and left arm, mimicking angina pectoris (11).

Barrett's esophagus is a premalignant condition in which metaplastic specialized columnar epithelium with gobble cells is present in the tubular esophagus (12). Barrett's esophagus is diagnosed with a combination of endoscopic and histologic criteria. The detection of intestinal metaplasia in biopsy samples taken from the abnormal-appearing esophageal mucosa in the distal esophagus is pathognomonic. Barrett's esophagus is detected at a frequency of 10–15% in endoscopy. It is an important risk factor for adenocancer development. Adenocarcinoma incidence in patients with BE is nearly 1% per year. Patients typically present with dysphagia and weight loss in the seventh or eighth decade (13).

Extraesophageal complications of GERD are also common in the elderly such as atypical noncardiac chest pain, laryngitis, sinusitis, otitis media, dental erosions, and pulmonary problems, such as aspiration pneumonia and chronic cough (14, 15). Diagnostic testing is similar to that in younger patients with GERD (16). However, an aggressive approach is recommended in contrast to younger patients because of the higher incidence of severe and life-threatening complications including BE and esophageal cancer (17). The initial diagnostic test should be endoscopy.

In this study, we intended to compare the reflux symptoms and endoscopy results in older patients and adults and discuss the findings in light of the current literature.

## Materials and Methods

### Study Design

This study was planned as a retrospective case-control study. Data were collected from two centers, which were chosen because of using the same device and diagnostic criteria. All patients aged 18 years and over, who underwent gastroscopy with a diagnosis of GERD between January 2017 and September 2020 were included and their files were retrospectively scanned. Patients aged 51–64 were excluded from the study in order to draw a sharper demarcation between the two groups; young and elderly. Patients with pregnancy and those with a history of previous gastric and esophagus surgery were also excluded. Heartburn and regurgitation were accepted as typical; and dysphagia, odynophagia, and burping as atypical reflux symptoms (18). All patients had been receiving proton pump inhibitors (PPIs) at standard doses for at least 1 month and had poorly responded to the therapy. Upper endoscopy indications of the patients were symptoms resistant to ppi therapy, determination of GERD etiology, and advanced age. The Fujinon gastroscopy device was used in endoscopic procedures. The Los Angeles classification is used as the current endoscopic staging for esophagitis (Table 1) (19). With the combination of endoscopic and histologic criteria, BE is defined as the condition in which metaplastic columnar epithelium replaces the stratified squamous epithelium that normally lines the distal esophagus. A BE diagnosis is made with the detection of both gastric and intestinal metaplasia in biopsy samples taken from the salmon-colored mucosa (20). The demographic characteristics and clinical data of the groups were compared.

**Table 1:** Current endoscopic staging for esophagitis (Los Angeles classification).

Grade A	5 mm mucosal damage in mucosal folds
Grade B	Damage >5 mm in mucosal folds but no continuity between folds
Grade C	Mucosal damage is continuous between 2 or more mucosal folds, but not all-round
Grade D	All-around mucosal damage (more than 75% of the esophageal lumen)

*The presence of an ulcer, stenosis, and Barrett's metaplasia should be stated separately at each stage*.

The study protocol was approved by the Antalya Training and Research Hospital Ethics Committee (2020-269).

### Statistical Analysis

Data are expressed as mean ± standard deviation (SD) for each of the measured parameters. A *p*-value below 0.05 was considered statistically significant. Statistical comparisons were made using Pearson's Chi-square (χ^2^) test or Fisher's exact test to compare the effects of sex and endoscopic or clinical reflux esophagitis severity.

## Results

Two hundred eighty-six patients aged over 65 years and 261 patients aged under 50 years, a total of 547 patients who presented to our clinic with reflux symptoms and underwent endoscopy were included in the study. The mean ages of the young and older groups were 38 ± 7.2 and 68.2 ± 2.5 years, respectively. The demographic data and comorbid diseases of the two groups are shown in Table 2. The number of male patients was significantly higher in both groups. The male/female ratio was 5/3 and 2/1 in the young and older groups, respectively. When comparing the sexes of the two groups, there was no statistically significant difference. When we compared the groups in terms of comorbid diseases such as asthma (8.4 vs. 16.2%, *p* = 0.05), cardiovascular disease (10.3 vs. 51.3%, *p* = 0.001), diabetes mellitus (4.5 vs. 28.6%, *p* = 0.001), and obesity (15.7 vs. 33.9%, *p* = 0.04), significantly higher rates of all diseases were noted in the older patients.

**Table 2:** Demographic data and comorbid diseases in the young and older groups with gastro-esophageal reflux disease.

**Variable**	**Young group**	**Older group**	***p*-Value**
	***n* = 261, 47.8%**	***n* = 286, 52.2%**	
	***n* (%) mean ± SD**	***n* (%) mean ± SD**	
Age (years)	38 ± 7.2	68.2 ± 2.5	
Sex			>0.5^*^
Male	166 (63.6%)	192 (67.1%)	
Female	95 (36.4%)	94 (32.9%)	
Comorbidity			
Asthma	22 (8.4%)	46 (16%)	0.646
Cardiovascular disease	27 (10.3%)	147 (51.3%)	0.646
Diabetes mellitus	12 (4.59%)	82 (28.6%)	0.293
Obesity	41 (15.7%)	97 (33.9%)	0.271

* *Pearson's χ^2^-test*.

All patients had been receiving PPIs at standard doses for at least 1 month at the time of endoscopy. However, the other medications of patients could not be noted because of the study's retrospective design.

The comparison of endoscopic reflux esophagitis severity and reflux symptoms of the groups is shown in Table 3. The young patients had a higher prevalence of typical symptoms, burning and burping (50.9 vs. 23%, *p* = 0.005 and 64.3 vs. 32.1%, *p* = 0.05, respectively), and atypical symptoms including hoarseness (25.6 vs. 18.1%, *p* = 0.05), dysphagia and/or odynophagia (50.2 vs. 20.9%, *p* = 0.001), and dry cough (30.2 vs. 21.3%, *p* = 0.01) than the older patients. However, another typical symptom, regurgitation, was higher in the older patients than in the young patients (38.6 vs. 43.7%, *p* = 0.01). When we examined the endoscopic findings in both groups, according to the Los Angeles classification, more severe esophagitis was found in older patients as compared with the young patients. As would be expected, hiatus hernia and esophageal cancer were also more common in the older patients. We found the esophageal cancer rates as 3.8 and 0.7% in the older patients and young patients, respectively (*p* = 0.001).

**Table 3:** The comparison of the reflux symptoms and endoscopic reflux esophagitis severity of the groups.

**Variable**	**Young group**	**Older group**	*p*-Value^*^
	**(*n* = 261, 47.8%)**	**(*n* = 286, 52.2%)**	
Reflux symptoms			
Burning	133 (50.9%)	66 (23%)	0.005
Regurgitation	101 (38.6%)	125 (43.7%)	0.05
Dysphagia and/or Odynophagia	131 (50.2%)	60 (20.9%)	0.001
Hoarseness	67 (25.6%)	52 (18.1%)	0.05
Burping	168 (64.3%)	92 (32.1%)	0.05
Dry cough	79 (30.2%)	61 (21.3%)	0.01
Esophagitis severity			
Normal	162 (62%)	82 (28.6%)	0.001
L.A. Grade A	50 (19.2%)	96 (33.5%)	0.01
L.A. Grade B	27 (10.2%)	50 (17.4%)	0.01
L.A. Grade C	15 (5.5%)	20 (7.1%)	0.05
Barret esophagus	7 (2.4%)	27 (9.6%)	0.001
	1 female, 6 males	8 females, 19 males	
Esophagus cancer	2 (0.7%)	11 (3.8%)	0.001
	2 females	3 females, 8 males	
Hiatus hernia			
Normal	222 (85.9%)	207 (72.4%)	0.05
<2 cm	22 (8.4%)	44 (15.4%)	0.005
2–5 cm	12 (4.6%)	23 (8%)	0.005
>5 cm	3 (1.1%)	12 (4.2%)	0.001
HH by sex	17 females, 20 males	44 females, 37 males	0.5

*L.A., Los Angeles classification; HH, hiatus hernia*.

* *Fisher's exact test*.

## Discussion

Reflux is a common disease in clinical practice. Although reflux prevalence is between 1 and 25% in the community, it has been reported with a frequency of 10.9% in men and 5.3% in women aged over 65 years (21). Degenerative changes in smooth muscles, lower esophageal sphincter pressure, and a decrease in peristalsis are seen anatomically in old age. Accordingly, an increase in the reflux of stomach contents to the esophagus is detected. This condition is usually with varying degrees of damage in the esophagial mucosa and symptoms. The most common symptoms of GERD are heartburn and acid regurgitation, which typically increases after meals, and there is usually relief after antacid therapy. The frequency of severe heartburn seems to decline with age, possibly due to a decrease in esophageal pain perception and atrophic gastritis (12).

It is known that GERD has more serious consequences and complications in the elderly compared with young patients. Furthermore, clinical features and typical symptoms of GERD are less common in older patients such as stomach pain, heartburn, and regurgitation (9). Dysphagia and abundant food regurgitation may be more prevalent in older patients, whereas typical retrosternal burning is a relatively rare symptom. Retrosternal burning and pain may not be felt even in stage C–D esophagitis due to changes in the perception threshold in older patients. Large hiatal hernias may be the most common benign cause of swallowing difficulty in the geriatric age group. In addition, comorbidities, especially neurological diseases (e.g., Parkinson's disease) or medication conditions such as the use of calcium antagonists should be taken into account in older patients.

Disease symptoms and disease severity are not always correlated in the elderly. Retrosternal burning seen in young people and specific for GERD may not always be observed in this age group. By contrast, older people often present with dysphagia, vomiting, anorexia, anemia, cough, and respiratory symptoms such as asthma. Esophagitis, typical reflux findings, retrosternal burning, and acid regurgitation may not be seen in the elderly in endoscopy. In addition, pain perception may decrease because of the increased gastric PH due to atrophic gastritis, which is common in the elderly (14).

In our study, in accordance with the literature, most of the typical symptoms were found to be higher in young patients as compared with the older patients, except for regurgitation, which was more common in the older patients than in young patients. However, the difference was not statistically significant. We thought that this situation might be related to hernia, which is more common in the elderly. Therefore, it can be concluded that the typical esophageal symptoms of GERD are not reliable markers for this disease in older patients.

Although hoarseness and dry cough are known as extra-esophageal symptoms of GERD, these are also more common in young patients than in the elderly. Thus, we can say that, in our study, the younger patients with GERD had more typical esophageal and extraesophageal symptoms than the older patients. In a study by Sidhwa et al., similar to our study, esophageal and extraesophageal symptoms were significantly higher in young patients (22). Elderly patients with GERD may have comorbid disease that can damage LES tonus. The medication(s) used for comorbid disease(s) including nitrates, calcium channel blockers, benzodiazepines, anticholinergics, and antidepressants may also induce reflux (23). As can be seen in Table 2, when comorbid diseases were evaluated, they were significantly more common in the older group. Although there are milder symptoms in older patients, the high rate of esophagitis may be related to this condition. Studies showing that lung and heart diseases and drugs used for these diseases trigger GERD support our idea (24, 25). However, although polypharmacy commonly used in the elderly population have been associated with GERD, unfortunately it was not possible to reach the medications of patients because of the study's retrospective design.

Although reported to be high in elderly (26), dysphagia and/or odynophagia were more common in young group in our study. It was thought that this situation might be related to high smoking and alcohol use, fast food diet, and irregular life habits (eating late, going to bed right after dinner) in the young patient group.

As can be seen in Table 3, when the severe forms of esophagitis such as grade C and grade D, BE and esophageal cancer were evaluated, there was a significant increase in the older group compared with the young group. As is known, BE often occurs in older ages and is detected during upper gastrointestinal endoscopy for various reasons. The male/female ratio is approximately 2:1 (27). In our study, this rate was found to be quite high as 2.7:1. However, the sex ratios presented for BE may vary with geographic location. Variable genetic susceptibility/protection across populations and differences in exposure to risk factors may be possible explanations for this situation (28).

As mentioned in the introduction, hiatus hernia is more common in older patients (29). In a recent review, the frequency of hiatus hernia was reported to increase with age; however, no sex effect has been defined, different series showed male predominance, female predominance, or no difference (21). In our study, we found no significant difference between hernia and sex (*p* = 0.5). However, there was a significant difference between the young and older groups. Hiatus hernias larger than 2 cm were found in 5.7% of young patients and 12.2% in the older patients (*p* = 0.01).

To date, there is no guidelines about the optimal method of GERD diagnosis in the elderly (30). As a new finding of the study, with higher prevalence of severe disease and complications, and presence of atypical symptoms or absence of symptoms, upper endoscopy should be encouraged earlier in the elderly, as it helps to determine the probable causes of GERD and avoids delay in diagnosis (17).

There are some strenghts and limitations of our study. The large number of patients is the main strenght. As for the limitations, some issues that increase reflux such as alcohol, smoking, eating habits, and obesity could not be taken into account because our study was retrospective. Used medications, except for PPIs, were not known, either. Again, due to our study's retrospective nature, patients' endoscopies were performed by different physicians, which may have led to differences in esophagitis and hernia scoring.

In conclusion, in our study, older and young adult patients with GERD had different characteristics. The older patients with GERD were predominantly male, rarely presented with typical GERD symptoms, and although their symptoms were milder, endoscopic findings are more serious.

## Data Availability Statement

The original contributions presented in the study are included in the article/supplementary material, further inquiries can be directed to the corresponding author/s.

## Ethics Statement

The studies involving human participants were reviewed and approved by Antalya Training and Research Hospital of Ethics Committee (16.09.2020-12/18).

## Author Contributions

BB and SG designed the study. BB, BP, SG, and HC performed the research. BP, SG, HC, and MU analyzed the data. BP, SG, and HC wrote the manuscript. BB and MU gave supervision and/or revised the manuscript. HA contributed to the design of the study and gave supervision and revised the manuscript during revision process. All authors have read and approved the submitted version.

## Conflict of Interest

The authors declare that the research was conducted in the absence of any commercial or financial relationships that could be construed as a potential conflict of interest.

## Publisher's Note

All claims expressed in this article are solely those of the authors and do not necessarily represent those of their affiliated organizations, or those of the publisher, the editors and the reviewers. Any product that may be evaluated in this article, or claim that may be made by its manufacturer, is not guaranteed or endorsed by the publisher.
